# Effects of *Sphagnum* Leachate on Competitive *Sphagnum* Microbiome Depend on Species and Time

**DOI:** 10.3389/fmicb.2019.02042

**Published:** 2019-09-06

**Authors:** Samuel Hamard, Bjorn J. M. Robroek, Pierre-Marie Allard, Constant Signarbieux, Shuaizhen Zhou, Tongchai Saesong, Flore de Baaker, Alexandre Buttler, Geneviève Chiapusio, Jean-Luc Wolfender, Luca Bragazza, Vincent E. J. Jassey

**Affiliations:** ^1^ECOLAB, Laboratoire d’Ecologie Fonctionnelle et Environnement, Université de Toulouse, CNRS, Toulouse, France; ^2^Laboratory of Ecological Systems (ECOS), Ecole Polytechnique Fédérale de Lausanne (EPFL), School of Architecture, Civil and Environmental Engineering, Lausanne, Switzerland; ^3^Swiss Federal Institute for Forest, Snow and Landscape Research (WSL), Lausanne, Switzerland; ^4^Laboratoire de Géologie, UMR 8538, CNRS-ENS, Ecole Normale Supérieure, Paris, France; ^5^School of Biological Sciences, University of Southampton, Southampton, United Kingdom; ^6^Aquatic Ecology and Environmental Biology Group, Faculty of Science, Institute for Water and Wetland Research, Radboud University, Nijmegen, Netherlands; ^7^School of Pharmaceutical Sciences, University of Geneva, University of Lausanne, Geneva, Switzerland; ^8^Department of Pharmaceutical Chemistry and Pharmacognosy, Faculty of Pharmaceutical Sciences and Center of Excellence for Innovation in Chemistry, Naresuan University, Phitsanulok, Thailand; ^9^Laboratoire Chrono-Environnement, Université Bourgogne Franche Comté, UMR CNRS 6249 USC INRA, Montbéliard, France; ^10^Laboratoire Carrtel, Université Savoie Mont Blanc INRA 042, Domaine Universitaire Belledonne, Le Bourget-du-Lac, France; ^11^Department of Life Science and Biotechnologies, University of Ferrara, Ferrara, Italy

**Keywords:** allelopathy and allelochemicals, metabolomics, microbial networks, microbial respiration and biomass, peatland, plant competition, plant-exudates, soil food-web

## Abstract

Plant specialized metabolites play an important role in soil carbon (C) and nutrient fluxes. Through anti-microbial effects, they can modulate microbial assemblages and associated microbial-driven processes, such as nutrient cycling, so to positively or negatively cascade on plant fitness. As such, plant specialized metabolites can be used as a tool to supplant competitors. These compounds are little studied in bryophytes. This is especially notable in peatlands where *Sphagnum* mosses can dominate the vegetation and show strong interspecific competition. *Sphagnum* mosses form carpets where diverse microbial communities live and play a crucial role in *Sphagnum* fitness by regulating C and nutrient cycling. Here, by means of a microcosm experiment, we assessed to what extent moss metabolites of two *Sphagnum* species (*S. fallax* and *S. divinum*) modulate the competitive *Sphagnum* microbiome, with particular focus on microbial respiration. Using a reciprocal leachate experiment, we found that interactions between *Sphagnum* leachates and microbiome are species-specific. We show that both *Sphagnum* leachates differed in compound richness and compound relative abundance, especially sphagnum acid derivates, and that they include microbial-related metabolites. The addition of *S. divinum* leachate on the *S. fallax* microbiome immediately reduced microbial respiration (−95%). Prolonged exposition of *S. fallax* microbiome to *S. divinum* leachate destabilized the food web structure due to a modulation of microbial abundance. In particular, leachate addition decreased the biomass of testate amoebae and rotifers but increased that of ciliates. These changes did not influence microbial CO_2_ respiration, suggesting that the structural plasticity of the food web leads to its functional resistance through the replacement of species that are functionally redundant. In contrast, *S. fallax* leachate neither affected *S. divinum* microbial respiration, nor microbial biomass. We, however, found that *S. fallax* leachate addition stabilized the food web structure associated to *S. divinum* by changing trophic interactions among species. The differences in allelopathic effects between both *Sphagnum* leachates might impact their competitiveness and affect species distribution at local scale. Our study further paves the way to better understand the role of moss and microbial specialized metabolites in peatland C dynamics.

## Introduction

Plant species composition and diversity play a fundamental role in regulating ecological processes such as nutrient and carbon (C) fluxes through their linkages with belowground components. Notably, plants are known to put a selective pressure on soil microbes and their activities (Robroek et al., 2015, 2017a; Cúcio et al., 2016; Sánchez-Cañ;izares et al., 2017), and to drive microbial assemblages in soil (Berg and Smalla, 2009; Schlatter et al., 2015; Tkacz et al., 2015). This selective effect is performed by plant-derived chemicals, either through the amount and form of carbon and other nutrients that plants provide to the soil (Huang et al., 2014; Cline and Zak, 2015; Sasse et al., 2018), or through specialized metabolites – i.e., allelochemicals – that directly interact with microbes (Bertin et al., 2003; Musilova et al., 2016; Niro et al., 2016). These plant-derived chemicals allow the establishment of mutual, competitive and defensive relationships between specific plants and microorganisms (Latif et al., 2017), and may have indirect effects on competing plants. Such effects could arise from either direct phytotoxic or stimulatory effects on the microbial community on which competing plants rely for nutrients uptake. This possibility is exemplified by plants that inhibit mutualistic bacteria or fungi that competitive plants require for optimal growth, but that the donor plant does not need (Cipollini et al., 2012). Allelopathy can also modify plant-microbe interactions by favoring growth of pathogenic or parasitic microbes that harm competitive plants (Cipollini et al., 2012; Geisen et al., 2018). Understanding the effects of plant-derived metabolites on the specific plant microbiome as a competitive mechanism is therefore of key importance to understand the dynamics of ecological processes within ecosystems.

The majority of studies on plant-microbial interactions focus on vascular plant-dominated ecosystems, largely ignoring the importance of cryptogams. Yet, cryptogams occur in many terrestrial habitats and contribute significantly to global ecosystem functions such as nitrogen and C cycle (Elbert et al., 2012). The processes linked to cryptogam metabolites (i.e., regulation of microbial activity, indirect plant competition) in cryptogam-dominated ecosystems remain poorly explored, while they could be rather important for ecosystem functions (Asplund et al., 2013; Asplund and Wardle, 2013). This is especially the case in ombrotrophic peatlands, where cryptogams such as *Sphagnum* mosses can dominate the ecosystem (Yu et al., 2011). *Sphagnum* mosses form expansive carpets that provide a habitat for a large diversity of microbial communities (Gilbert et al., 1998; Jassey et al., 2013, 2015; Bragina et al., 2014; Mieczan et al., 2015a, b). The *Sphagnum* microbiome is structured in a microbial food web constituted by bacteria, fungi (decomposers), protists (producers, predators, top-predators), and small-sized metazoan (predators, top-predators). The functioning of this *Sphagnum-*associated food web critically determines the cycling of C and nutrients (Jassey et al., 2015) through the microbial loop (Gilbert et al., 1998) –a trophic pathway through which C and nutrients from organic matter are returned to higher trophic levels through their incorporation into bacterial and fungal biomasses. Empirical observations suggest that the structure and activity of the *Sphagnum* microbiome determine *Sphagnum* fitness (Kostka et al., 2016). For instance, microbial functional guilds such as methanotrophic bacteria, photosynthetic protists and nitrogen-fixing cyanobacteria clearly benefit the host-species by providing a source of C and/or nitrogen that enhances moss productivity (Jassey et al., 2015; Kostka et al., 2016; Carrell et al., 2019). Hence, it can be argued that any shift in the composition of the *Sphagnum* microbiome can modulate *Sphagnum* productivity and, ultimately, ecosystem C cycling.

Different species of *Sphagnum* coexist in peatlands. Each species has a specific productivity and performance (Gunnarsson, 2005; Robroek et al., 2007), and each species is associated to a specific microbiome (Opelt et al., 2007; Bragina et al., 2012). It has been observed that *Sphagnum* species often grow in spatially-structured population in response to interspecific competition (Ingerpuu and Vellak, 2013). Apart from environmental effects such as water-table level or niche separation (Bragazza, 1997; Robroek et al., 2007), the mechanisms that drive competition are not well determined. *Sphagnum* exudates can affect the growth of other *Sphagnum* species (Ingerpuu and Vellak, 2013), suggesting that *Sphagnum* metabolites may play a role in interspecific competition. This hypothesis is supported by the fact that *Sphagnum* mosses produce a variety of specialized metabolites (Rasmussen et al., 1995; Opelt et al., 2007; Chiapusio et al., 2018), such as phenolic acid derivatives (Rudolph and Samland, 1985; Rasmussen et al., 1995), with potential allelopathic effects (Verhoeven and Liefveld, 1997). Phenolic acids, particularly sphagnum acid, are highly water-soluble compounds (Rasmussen et al., 1995) and have been long suspected of antimicrobial effects in peatlands (Verhoeven and Liefveld, 1997; Binet et al., 2017). Recent studies further suggest that polyphenol compounds could be an important factor modulating the structure of microbial assemblages in *Sphagnum* peatlands (Jassey et al., 2011b, c, 2013). Altogether, these studies indicate an important possible role of *Sphagnum* exudates in affecting peatland microbial activity and suggest their potential implication in interspecific *Sphagnum* competition by alteration of the microbiome, ultimately affecting *Sphagnum* fitness, peatland primary productivity and the peatland C cycle. Understanding the role of *Sphagnum* exudates in driving microbial communities is thus crucial to better understand peatland C dynamics.

Here we focus on two widespread *Sphagnum* species, i.e., *S. fallax* and *S. divinum*, that often co-occur together, to assess whether *Sphagnum* interspecific competition and ecosystem functioning can be indirectly mediated by a shift in their respective microbiome. We tested how each of these two *Sphagnum* species affected the structure and the functioning of the microbial community associated to the competitive species. Using a reciprocal leachate-transfer experiment, we assessed whether a brief exposition to allochthonous *Sphagnum* leachates (i.e., leachates from the competing species) altered the CO_2_ respiration of the microbiome. Second, we tested if a prolonged exposition to allochthonous *Sphagnum* leachates altered the microbial food-web structure and functioning, emphasizing on microbial activity and C related processes. We hypothesized that: (1) a short-term exposure to allochthonous leachates will inhibit microbial respiration due to species-specific metabolites inhibitory effects, and that this effect is universal across the two species; (2) a prolonged exposure to allochthonous leachate will alter the structure of microbiome causing a decrease of microbial metabolism.

## Materials and Methods

### Field Sampling and Leachate Collection

In April 2015, we collected 15 intact shallow cores (diam. 11 cm; depth 15 cm) of *Sphagnum fallax* and *S. divinum* (30 cores in total) in the Store Mosse National Park, Sweden (57°17′54 N, 14°00′39 E, permit 521-895-2011). Cores were extracted in a habitat that was low in vascular plant cover (<5%), but occasional specimens were carefully removed after which the cores were placed in PVC pipes (hereafter referred as microcosms) that were open from the top and closed at the bottom. Microcosms were then transported to the laboratory facilities at the Ecole Polytechnique Fédérale de Lausanne, Switzerland, were they were kept in a growth chamber (20°C, 70% RH, 14 h/10 h day/night photoperiod, PAR intensity 200 μmol m^–2^ s^–1^) for 2 weeks to acclimate. During the acclimation period, all microcosms were watered daily with 12 mL of artificial rainwater (Garrels and Christ, 1965), which sufficed to keep water levels at field conditions (−1 cm for *S. fallax* and −3 cm for *S. divinum*). During the acclimation period, we collected the leachates from all microcosms. To do so, we first drained the microcosms and added fresh artificial rainwater. This was repeated after 2 days, with the difference that this time all leachate was collected. Leachates from microcosms with the same species were combined in a bulk leachate, resulting in one leachate for *S. fallax* (L_*SF*_) and one for *S. divinum* (L_*SD*_). After collection, both leachates were filtered at 0.2 μm to remove microorganisms, and frozen (−20°C) until utilization.

### Leachate Chemical Characterization

After leachate collection, a set of chemical analyses were performed for both L_*SF*_ and L_*SD*_ leachates. The concentrations of dissolved organic carbon (DOC) and nitrogen (DON) were quantified by combustion using a Shimadzu analyzer (TOC-V CPH). The quality of DOC was determined by spectroscopy analysis by measuring the absorbance within the range of wavelengths 250–665 nm (Jaffrain et al., 2007). Spectral slopes (S_250__–__665_, nm^–1^) were calculated using linear least squares regressions with Ln-transformed absorptions. High S_250__–__665_ values indicate low molecular weight material and/or decreasing aromaticity of the leachate (Hansen et al., 2016). Leachate phenolic content was determined using the Folin and Ciocalteu’s reagent with gallic acid as standard (Jassey et al., 2011b). Phenolic content was expressed as mg of equivalent gallic acid per volume of leachate (mg eq. gallic acid.L^–1^).

A detailed chemical analysis of the metabolites present in *Sphagnum* leachates, *Sphagnum* mosses and bog water was performed using Ultra-High Pressure Liquid Chromatography coupled with High Resolution Mass Spectrometry (UHPLC-HRMS; see details below). To disentangle the provenance of the metabolites (*Sphagnum* versus peat), several comparisons were made. First, we compared leachate metabolic composition to water and methanolic extracts of lyophilized *S. fallax* and *S. divinum* (Jassey et al., 2011a). These extracts were prepared by grinding 0.05 g dry weight of lyophilized *Sphagnum* (0–3 cm; capitulum) in 5 mL of water (mili-Q) or methanol (80/20 methanol/distilled water v/v) using metal ball grinder. We also compared leachate metabolic profiles to the one of the bog water as comparison. *S. fallax*, *S. divinum* and bog water were sampled in the Store Mosse site during the core sampling campaign. All samples were filtered through SPE columns, and directly injected in the UHPLC-HRMS. In total, we analyzed seven metabolic profiles through UHPLC-HRMS: the bog water, two *Sphagnum* leachates, two *Sphagnum*-water extracts, and two *Sphagnum*-methanolic extracts.

#### UHPLC-HRMS Analysis

We qualitatively assessed the metabolic composition of both leachates using High-resolution Mass Spectrometry (MS) and molecular networking. Briefly, chromatographic separation was performed on a Waters Acquity UPLC system interfaced to a Q-Exactive Focus mass spectrometer (Thermo Fisher Scientific, Bremen, Germany), using a heated electrospray ionization (HESI-II) source. Thermo Scientific Xcalibur 3.1 software was used for instrument control. The LC conditions were as follows: column, Waters BEH C18 50 × 2.1 mm, 1.7 μm; mobile phase, (A) water with 0.1% formic acid; (B) acetonitrile with 0.1% formic acid; flow rate, 600 μL.min^–1^; injection volume, 1 μL; gradient, linear gradient of 2–100% B over 6 min and isocratic at 100% B for 0.6 min. An Acquity IClass UPLC photodiode array detector was used to acquire PDA spectra, which were collected from 210 to 450 nm. In positive ion mode, diisooctyl phthalate C_24_H_38_O_4_ [M + H]^+^ ion (*m/z* 391.28429) was used as internal lock mass. The optimized HESI-II parameters were as follows: source voltage, 4.0 kV (pos); sheath gas flow rate (N2), 55 units; auxiliary gas flow rate, 15 units; spare gas flow rate, 3.0; capillary temperature, 275.00°C (pos), S-Lens RF Level, 45. The mass analyzer was calibrated using a mixture of caffeine, methionine–arginine–phenylalanine–alanine–acetate (MRFA), sodium dodecyl sulfate, sodium taurocholate, and Ultramark 1621 in an acetonitrile/methanol/water solution containing 1% formic acid by direct injection. The data-dependent MS/MS events were performed on the three most intense ions detected in full scan MS (Top3 experiment). The MS/MS isolation window width was 1 Da, and the stepped normalized collision energy (NCE) was set to 15, 30, and 45 units. In data-dependent MS/MS experiments, full scans were acquired at a resolution of 35,000 FWHM (at *m/z* 200) and MS/MS scans at 17 500 FWHM both with an automatically determined maximum injection time. After being acquired in a MS/MS scan, parent ions were placed in a dynamic exclusion list for 2.0 s.

#### LC-MS Data Processing

ThermoRAW MS data were converted to the open MS format (.mzXML) using the MSConvert software, part of the ProteoWizard package (Chambers et al., 2012). The converted files were then treated using the MzMine 2.36 software suite. The parameters were adjusted as follow: the centroid mass detector was used for mass detection with a noise level set to 1.0E6 for MS level set to 1, and to 0 for MS level set to 2, respectively. The ADAP chromatogram builder was used and set to a minimum group size of scans of 5, minimum group intensity threshold of 1.0E5, minimum highest intensity of 1.0E5 and *m/z* tolerance of 8.0 ppm. For chromatogram deconvolution, the algorithm used was the wavelets (ADAP). The intensity window S/N was used as S/N estimator with a signal to noise ratio set at 25, a minimum feature height at 10,000, a coefficient area threshold at 100, a peak duration ranges from 0.02 to 0.9 min and the RT wavelet range from 0.02 to 0.05 min. Isotopes were detected using the isotopes peaks grouper with a *m/z* tolerance of 5.0 ppm, a RT tolerance of 0.02 min (absolute), the maximum charge set at 2 and the representative isotope used was the most intense. An adduct (Na^+^, K^+^, NH_4_^+^, ACN^+^, CH_3_OH^+^, Isopropanol^+^) search was performed with the RT tolerance set at 0.1 min and the maximum relative peak height at 500%. A complex search was also performed using [M + H]^+^ for ESI positive mode, with the RT tolerance set at 0.1 min and the maximum relative peak height at 500%. A custom database search was finally performed using the Dictionary of Natural Products 2018 (v. 26.2) database^1^, restricted to moss metabolites. Peak alignment was performed using the join aligner method (*m/z* tolerance at 8 ppm), absolute RT tolerance 0.065 min, weight for *m/z* at 10 and weight for RT at 10. The peak list was gap-filled with the same RT and *m/z* range gap filler (*m/z* tolerance at 8 ppm). Eventually the resulting aligned peaklist was filtered using the peak-list rows filter option in order to keep only features associated with MS2 scans. Full parameters are available as .xml file as supporting information (Sphagnol_profiles_MzMineparams.mzmine).

#### LC-MS Data Analysis: Molecular Networks Generation

In order to further identify compounds in MS dataset, we used the molecular networking (MN) approach that group metabolites by structural similarity (Wang et al., 2016). The MN approach is based on the organization and visualization of tandem MS data through a spectral similarity map, revealing the presence of similar MS fragmentations patterns. As structurally related compounds tend to share similar fragmentation spectra, nodes gathered together create clusters of structural analogs. The generated molecular networks were annotated using experimental spectral libraries (GNPS libraries^2^) and an *in silico* fragmented database of natural products using a previously detailed dereplication strategy (Allard et al., 2016). In the network created, edges were filtered to have a cosine score above 0.65 and more than 6 matched peaks. Further edges between two nodes were kept in the network if and only if each of the nodes appeared in each other’s respective top 10 most similar nodes. The spectra in the network were then searched against GNPS’ spectral libraries. The library spectra were filtered in the same manner as the input data. All matches kept between network spectra and library spectra were required to have a score above 0.7 and at least 6 matched peaks. The output was visualized using Cytoscape 3.6 software^3^. The nodes of the generated networks were colored according to the number of MS/MS triggered in a given sample, thus offering a semi-quantitative information. The size of the nodes was mapped according to the total sum of precursor ions intensities. Molecular networks are available on the GNPS servers at the following addresses: https://gnps.ucsd.edu/ProteoSAFe/status.jsp?task=00de48872c8b4d76b108a40fdfb7ea0a and https://gnps.ucsd.edu/ProteoSAFe/status.jsp?task=60f9f10962ae4aa0952a54c9eb9fc21d.

### Experimental Setup and Measurements

To assess the allelopathic effects of *Sphagnum* leachate addition on potential neighboring competing species, we set up a targeted reciprocal leachate experiment. We first divided the species-specific mesocosms into two experimental groups. One group, consisting of 10 microcosms (five per species), was used to test the instantaneous effect of allochthonous leachate addition on the *Sphagnum* microbiome. The second group, consisting of 20 microcosms (10 per species), was used to test the effect of prolonged exposure to allochthonous leachates.

#### Instantaneous Leachate Effects

We sampled 3 g of *Sphagnum* shoots (0–4 cm depth) from all microcosms and placed them in falcon tubes (*n* = 5). From these shoots, we extracted the microbiome by shaking at 40 rpm for 1.5 h in 30 mL of Mili-Q water^®^. The extracted microbiome was recovered by filtration at 1 mm. For each microcosm, 0.8 mL of microbial extract was transferred to a Microresp^TM^ (Campbell et al., 2003) 96-deep-well microplate, allocating 8 wells to each microcosm. Subsequently, we added 0.2 mL artificial rainwater solution (+ water) to four of these technically replicated microbiomes while the other four microbiomes received 0.2 mL of allochthonous leachates (+ leachate). On the whole, this resulted in four incubation treatments, i.e., two for *S. fallax* microbiome (SF microbiome + water and SF microbiome + SD leachate), and two for *S. divinum* microbiome (SD microbiome + water and SD microbiome + SF leachate). After substrate addition (rainwater or allochthonous leachates), the 96-deep-well microplate was sealed with a 96-well detection microplate containing agar gel and cresol red as indicator dye (Campbell et al., 2003), and incubated at 20°C in the dark. Discoloration of the indicator gel was measured using spectroscopy at 570 nm at irregular intervals (BioTek SynergyMX). The absorbance values were normalized at a given time by the initial absorbance values, after which the percentage of CO_2_ released from each well was calculated (Campbell et al., 2003). Values for the four technical replicates were averaged after data-quality check.

In order to understand the effects of leachate composition on microbial respiration, we focused on the effects of *S. divinum* compounds on *S. fallax* microbial CO_2_ respiration. We used a fractionation of methanolic extracts of *S. divinum*. Methanolic extracts presented the advantage to be more concentrated than leachates which made the fractionation possible. 3.27 g of the methanolic extract of *S. divinum* were separated by Flash chromatography. Chromatographic conditions: PuriFlash^®^ C18 HQ column (15 μm particle size, 120G), gradient H_2_O (+0.1% FA)/MeOH (+0.1% FA) (25:75–100:0 in 109 min, 100:0 isocratic from 109 to 164 min), 2 μL (5 mg/mL), flow rate 30 mL/min, UV detection 200, 254, 280, and 366 nm, 18 mL fractions. The 136 resulting fractions were pooled in 23 fractions according to their thin layer chromatography profiles. The 23 fractions were dissolved in dimethyl sulfoxide (DMSO) at a concentration of 10 μg.L^–1^. As a next step, we incubated 0.8 mL of *S. fallax* microbial extract with the 0.2 mL of all obtained *S. divinum* metabolite fractions using an identical approach as aforementioned (Microresp^TM^). Microbial incubation with DMSO was used as a control. Following the same protocol as previously described, we assessed microbial respiration at irregular intervals.

#### Prolonged Effect of Leachates

Ten microcosms from each species were randomly divided into two groups. The first group (*n* = 5) was watered daily with 12 mL artificial rainwater, and hence served as a control (C). The second group received daily 12 mL of allochthonous leachate; *S. fallax* was watered with 12 mL of L_*SD*_ and *S. divinum* was watered with 12 mL of L_*SF*_. Essentially this resulted in four experimental treatments: SF-C, SF-L_*SD*_, SD-C, and SD-L_*SF.*_ The experiment lasted 3 weeks. Due to experimental constraints (i.e., limited leachate availability) we were not able to apply autochthonous leachate addition. The experiment was achieved over a 3 weeks period, which guarantees the microbiome to have turned over multiple times (Schönborn, 1965; Schmidt et al., 2007). Throughout the experiment, the position of the cores in the growth chamber was spatially randomized and we kept the same conditions applied during the acclimation period.

##### Microbial biomass and community structure

At the end of the 3 weeks leaching experiment, phospholipids fatty acids (PLFA) biomarkers were used to estimate the biomass of fungi, gram-negative, gram-positive, and actinomycete bacteria (Denef et al., 2009). PLFA were extracted from 0.25 g lyophilized *Sphagnum* shoot over a 12 h period in a solvent phase comprising 3.0 mL 50 mmol.L^–1^ phosphate buffer (pH 7.0), 3.8 mL chloroform and 7.6 mL methanol (Börjesson et al., 1998). PLFA 19:0 (Larodan, Malmö, Sweden) was added as an internal standard to the phospholipid fraction. PLFA were methylated to form fatty acid methyl esters using 1 mL of 0.2 mol.L^–1^ methanolic KOH (Sundh et al., 1997; Chowdhury and Dick, 2012) and analyzed on a gas chromatograph coupled to a mass spectrometer. Results were expressed as micrograms of PLFA per gram of *Sphagnum* dry mass (μg PLFA.g^–1^ DM).

The biomass of predators (bacterivores, fungivores, and omnivores) such as ciliates, testate amoebae, rotifers and nematodes was estimated using inverted microscopy (Olympus IX71, × 400, Utermöhl method). To this aim, we collected 3 g of fresh *Sphagnum* shoots (0–4 cm depth), fixed them in 20 mL of glutaraldehyde (2% final concentration) and stored at 4°C in the dark before analyses. Testate amoebae, ciliates, rotifers, and nematodes were extracted from mosses following the method described in Jassey et al. (2011b). From a 3 mL subsample, we identified microbial predators with the appropriate taxonomic literature (Lynn, 2006). The 3 mL aliquots were settled for 1 h in the counting chamber, which was more than enough according to ciliate sinking rates (Claessens and Prast, 2008). Cells were identified and enumerated across 50% of the total chamber area using transects. The abundance of each species was converted into biovolume (μm^3^), based on geometrical shapes and dimensions measured under the microscope (length or diameter; width, and height) and then into biomass using conversion factors as given in Gilbert et al. (1998). The biomass data were expressed as micrograms of C per gram of *Sphagnum* dry mass (μgC g^–1^ DM).

##### Microbial respiration and enzyme activity

We extracted the microbial communities from each of the 20 microcosms as described above. We incubated the microbial extracts in MicroResp^TM^ with rain water for 8 h, following the previous protocol. Microbial enzyme activity was quantified in microplates following Jassey et al. (2016). Fluorescence of fluorescein diacetate (FDA), a proxy for total enzyme activity (Green et al., 2006), was monitored spectrophotometrically with an excitation wavelength of 365 nm and emission detection at 450 nm (BioTek, SynergyMX). Spectrophotometric measurements were made every hour during incubation until a plateau was reached. We based the calculation of enzyme activities on incubation times of 3 h for FDA hydrolysis. Methodological controls containing boiled enzyme extracts were further assayed. Overall enzyme activity was then calculated by subtracting the mean fluorescence of boiled controls from the mean fluorescence of extracts wells. FDA enzyme activity was converted into μmoles per gram dry weight per min (μmol min^–1^ g^–1^ DM).

### Statistical Analyses

All statistical analyses were performed in R version 3.5.0 (R Core Team, 2018). Analysis of variance (ANOVA) was applied to test the effects of *Sphagnum* species, leachate treatments and their interaction (fixed effects) on CO_2_ release, microbial enzyme activity and microbial biomasses. Prior analyses, we checked the normality and the homoscedasticity of the data; the data were log-transformed when necessary. For time-related measurements (kinetics of microbial respiration) a linear mixed model was used with time, species and treatment as fixed variables and specific microcosm in function of time as a random effect (Pinheiro and Bates, 2000). Similar models were used to assess the effect of each *S. divinum* fraction on microbial respiration. Differences among metabolite relative abundance in both leachates were tested using chisquared-test.

### Food Web Numerical Analyses

#### Food Web Constructions

To elucidate the effect of prolonged leachate addition on microbial interactions within the *Sphagnum* microbiome, we used a network approach based on the force of trophic interactions among microbial species. For each treatment (i.e., SF-C, SF-L_*SD*_, SD-C, SD-L_*SF*_), we built an average food web based on the observations from the five replicates. These networks were built in four steps. Firstly, we assigned every species or generic group assessed with PLFA (i.e., gram-positive and gram-negative bacteria, actinomycete, other bacteria, saprophytic fungi, arbuscular mycorrhiza, and diatoms) to a trophic group: decomposers, primary producer, consumer and top-predator. We then identified the feeding habit of consumers and top-predators (Supplementary Table S1) using microscope observation and literature (Gilbert et al., 2000, 2003; Mieczan, 2009; Wilkinson and Mitchell, 2010; Jassey et al., 2012; personam observations). Based on these feeding habits, we constructed a general table of hypothetical trophic links between microbial species and/or groups. We obtained two tables of hypothetical links, one associated to *S. fallax* microbiome, the other associated to *S. divinum* microbiome. Secondly, we used basic rules to transform the general tables of hypothetical links into microcosm-specific tables of effective trophic links. A link between a predator and a prey was considered to be effective in a microcosm (i) if the link existed in the general table of hypothetical links, (ii) if the predator and the prey coexisted in microcosm, and (iii) if the predator was less abundant than the prey. Based on previous observations in peatlands (Jassey et al., 2013, 2015; Reczuga et al., 2018), we assumed that predators were always less abundant than bacteria, fungi and algae quantified by PLFA. Thirdly, we weighted the effective trophic links between a predator and a prey by the relative abundance of the predator amongst microcosms of a *Sphagnum* species to take into account the intensity of interactions. For instance, an effective link between a predator and a prey was considered more intense in microcosm A than in microcosm B if the predator was more abundant in microcosm A compared to microcosm B, both microcosms belonging to the same *Sphagnum* species. Finally, we built an average table of links for each treatment by averaging the microcosm-specific tables of weighted links according to their treatments. We only kept links with a weight higher than 0.2 in order to remove weaker interactions (Supplementary Figure S1), assuming that they were unlikely. Such threshold resulted in the removal of *c.* 20% of weaker links in *S. fallax* and *S. divinum* microcosms.

#### Food Web Structure Analyses

Networks were then produced using the *igraph* R package (Csárdi and Nepusz, 2006). We extracted the core properties of the webs to evaluate whether substructures responded to leachate addition according to Ma et al. (2019). Each network was then analyzed in terms of connectivity (C), density of links within the network (edge D), core size and density of links within the core (Phir). Connectivity was calculated as the number of links divided by the square of the number of species in the network. Density of links within the network was calculated as the number of effective links divided by the number of total possible links. Core size and density of links within the core were calculated as in Ma et al. (2019). Indices of network beta diversity were also calculated between networks (Poisot et al., 2012) using betalink *R* package (Poisot et al., 2016). Especially, we calculated network beta diversity between average and hypothetical networks (constructed with hypothetical trophic links as explained above) to see if treatments altered the distance with hypothetical networks.

#### Food Web Robustness Analyses

We tested the robustness and specificity of each network using a series of null models (Robroek et al., 2017b; Ma et al., 2019). We used three scenarios to simulate new networks. The first scenario (hereafter R_*I*_) comprised 1000 randomizations of trophic links among microbes whilst keeping the total number of links and species within network intact. The second scenario (hereafter R_*R*_) comprised the removal of trophic links of two species taken randomly, and is based on the observed effect of leachate addition on food web structures (see Results for details). Species removal was repeated until the whole combinations of species removal was reached (i.e., 231 combinations). The last scenario (hereafter R_*IR*_) was a combination between R_*I*_ and R_*R*_: 30 combinations of two species were randomly chosen based on our observations. For each combination, the links of the two species were removed before proceeding to 100 randomizations of the trophic links within the networks as described above (3000 reshuffled networks in total). On each simulated networks, we calculated structural indices (beta diversity, C, edgeD, Coresize, and Phir) and compared them with the structural indices found in original networks from leachate addition treatments using standardized effect size as described in Robroek et al. (2017b). Finally, we proceeded to a targeted species removal within networks based on species that dramatically changed their network position between control and leachate addition treatments (*Hyalosphenia papillo* and *Hyalosphenia elegans* in *S. fallax* networks and *Assulina muscorum* and *Hyalosphenia papillo* in *S. divinum* networks). Starting from the control networks, we proceeded to a target removal of these species when they were brought to network periphery in leachate addition treatments. On the contrary, we proceeded to a replacement of their effective links by their hypothetic links when they were brought to the heart of networks in leachate addition treatments. Again, we measured structural network indices and compared them with the observed structural indices.

## Results

### Global Chemical Characterization of *Sphagnum* Leachate

Leachate composition was species-specific with global compounds being more concentrated in L_*SF*_ than in L_*SD*_: water-soluble phenolic concentration was higher in L_*SF*_ (4.87 mg L^–1^) than in L_*SD*_ (3.92 mg L^–1^) as well as DOC (L_*SF*_ = 39 mg L^–1^; L_*SD*_ = 11.55 mg L^–1^ in) and DON (L_*SF*_ = 1.95 mg L^–1^; L_*SD*_ = 0.89 mg L^–1^). The spectral slope (S_250__–__665_) of DOC was lower in L_*SF*_ (0.067) than in L_*SD*_ (0.144), which indicates that the aromaticity and/or molecular weight of L_*SF*_ was higher than L_*SD*_.

**FIGURE 1:**
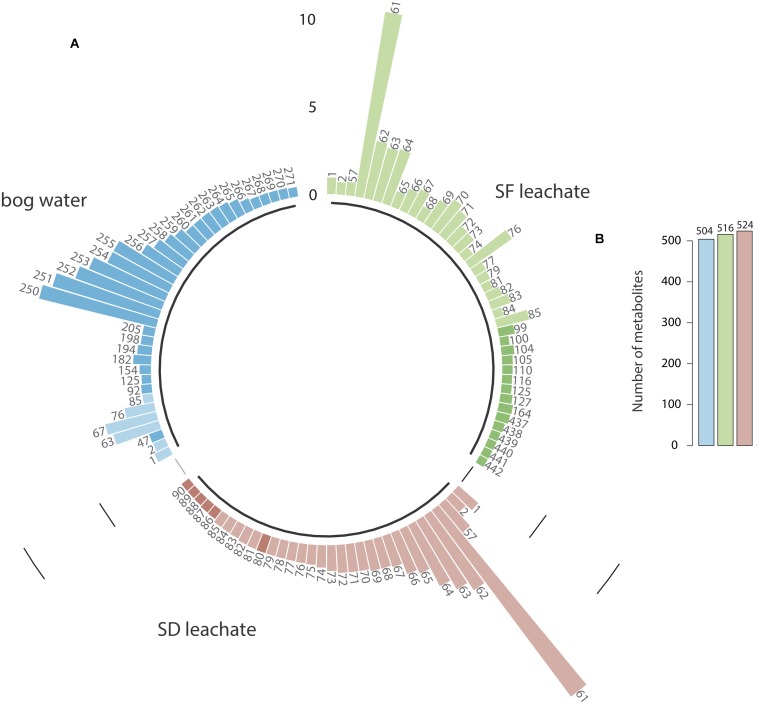
Relative abundance of metabolites in SF, SD leachates and bog water **(A)**. Each bar and number correspond to a specific metabolite. For clarity of the graph, only the more concentrated metabolites are presented (relative abundance >2.5%). Dark-colored bars refer to metabolites that have a relative abundance higher than 2.5% in the considered extract, but less than 2.5% in other extracts. Total number of metabolites in SF, SD leachates and bog water are presented in **(B)**.

According to the metabolite composition, we found that L_*SF*_ and L_*SD*_ contained almost the same chemical compounds but in different relative proportions (Figure 1A). L_*SD*_ (524 metabolites) was slightly richer in metabolites than L_*SF*_ (516 metabolites); 2% of these leachate-metabolites being species-specific, some of them being present only in L_*SD*_ (Supplementary Figure S2). Despite similar metabolite richness (Figure 1B), nearly half of the more concentrated compounds (relative abundance > 2.5%) differed between L_*SF*_ and L_*SD*_ (*P* < 0.01, Chi-test; Figure 1A). Furthermore, only 25% of leachate-metabolites were common with bog water metabolites. This comparison indicates that bog water and *Sphagnum*-leachates composition is different in terms of molecular compounds. The relative abundance of shared metabolites between *Sphagnum* leachates and bog water also differed (*P* < 0.01, Chi-test) (Figure 1A). Finally, we found according to the molecular network (Supplementary Figure S3) that *Sphagnum* leachates were closely related to water and methanolic *Sphagnum* extracts, which indicates that the metabolites from *Sphagnum* leachates are mostly released by *Sphagnum* mosses and do not come from the peat or vascular plants.

**FIGURE 2:**
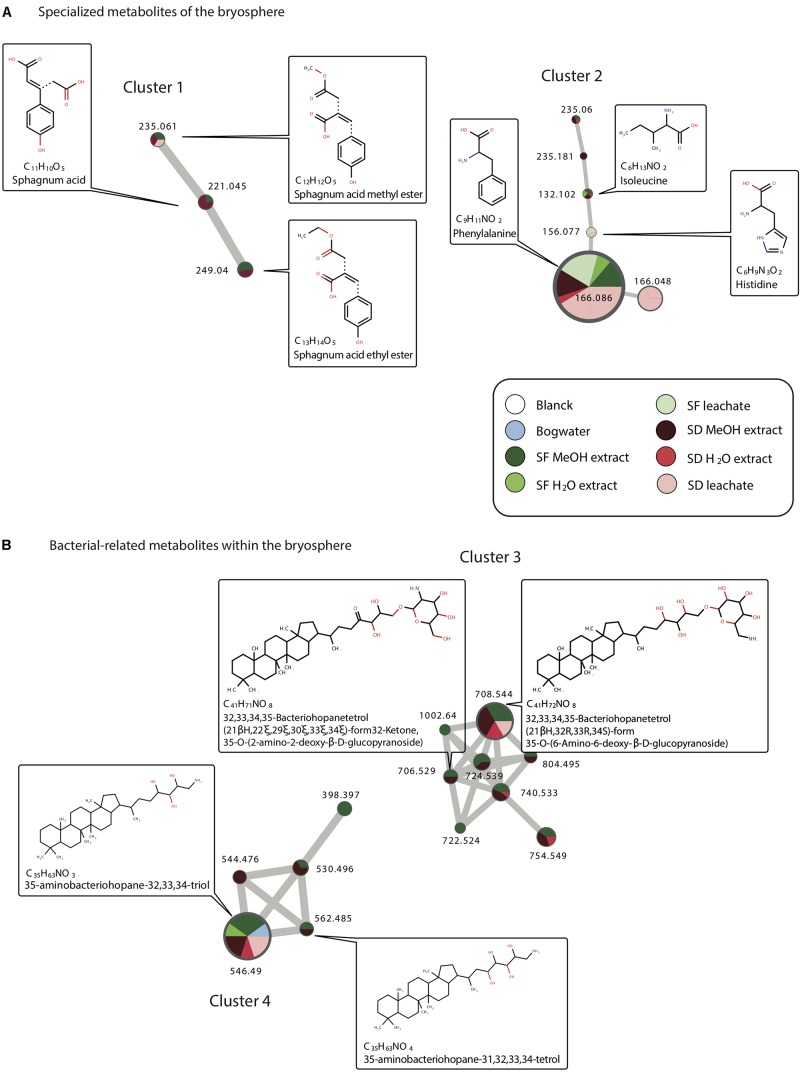
Clusters individualized from the molecular network (see Supplementary Figure S3) and showing identified specialized metabolics found in *Sphagnum* leachates, extracts, and bog water. Metabolites are associated to the bryosphere **(A)** or to bacteria **(B)**.

Focusing on metabolites common to methanolic/water extractions and leachates profiles, several annotated metabolites corresponded to aminoacids derivatives. Phenylalanine for example was found to be present in both *Sphagnum* species extract (water, methanolic) and leachates but not in bog water. Typical *Sphagnum* metabolites such as sphagnum acid were found to be present in water and methanolic extracts but not in leachates (Figure 2A). We however detected a derivate of sphagnum acid, sphagnum acid methyl ester, in L_*SD*_ but not L_*S*__*F*_. Finally, we also detected bacteria-related metabolites such as aminobacteriohopane and bacteriohopanetetrol derivatives (Figure 2B). 35-aminobacteriohopane-32,33,34-triol was found to be present in methanolic, water extracts of both species, in the bog water, but only in L_*SD*_. Another one [32,33,34,35-Bacteriohopanetetrol (21βH,32R,33R,34S)-form 35-O-(6-Amino-6-deoxy-β-D-glucopyranoside)], was detected in leachates, and water/methanolic extracts of *S. divinum* but only observed in the *S. fallax* methanolic extract (Figure 2B).

### Instantaneous Effect of an Allochthonous Leachate Addition on Microbial Respiration

**FIGURE 3:**
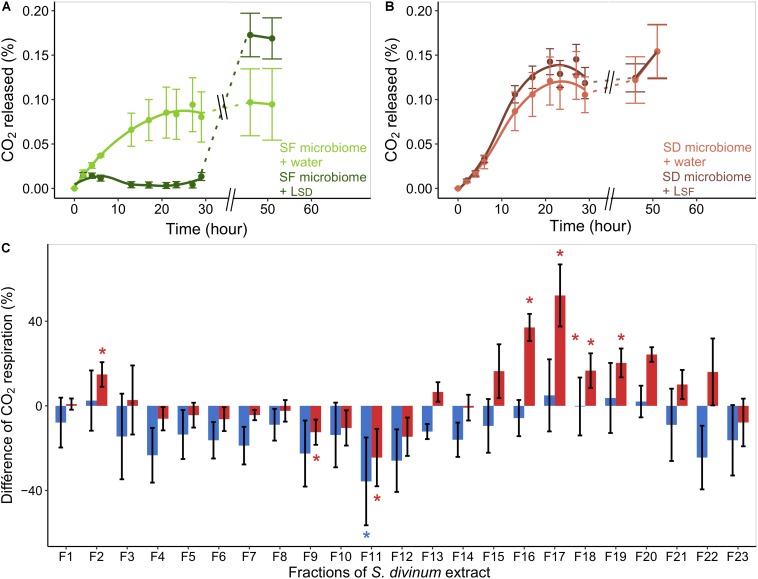
Response of microbial respiration to short-term allochthonous leachate addition. The CO_2_ released in each well is calculated by normalizing with the initial value and combined according to the treatment. *S. fallax* microbiome **(A)** were either incubated with water (lightgreen) or with L_*SD*_ leachate (darkgreen). Similarly, *S. divinum* microbiome **(B)** was either incubated with water (lightred) or with L_*SF*_ (darkred). Error bars refer to standard error. **(C)** Shows the difference in respiration of *S. fallax* microbiome after incubation with different fractions of L_*SD*_ compared to DMSO only (control). Differences are expressed as percentage. Blue bars show differences of respiration after 6 h of incubation and red bars after 21 h of incubation. Error bars refer to standard error and asterisks indicate significant differences with control for each incubation time (*P* < 0.05).

Allochthonous leachates addition effects on *Sphagnum* microbiome CO_2_ respiration are time and species-specific [*F*_(__1__, 173__)_ = 3.73, *P* = 0.05]. The respiration of *S. fallax-*associated microbiome was strongly inhibited by L_*SD*_ addition and reached only 5% of the control rates within the first 30 h [*F*_(__1__, 8__)_ = 10.38, *P* = 0.018; Figure 3A]. This inhibition was, however, time-limited so that after 46 h of incubation microbial respiration recovered and even released 78% more CO_2_ than controls [*F*_(__1__, 8__)_ = 3.8, *P* = 0.10]. We did not find an effect of L_*SF*_ on the microbial respiration of *S. divinum* microcosms [*F*_(__1__, 81__)_ = 0.56, *P* = 0.48; Figure 3B].

The incubation of *S. fallax* associated microbiome with *S. divinum* metabolic fractions as substrate confirmed the inhibition of microbial respiration on short-term. After 6 h of incubation, *S. divinum* metabolic fractions reduced *S. fallax* microbial respiration by 12% on average compared to the DMSO control (Figure 3C). We found that 19 fractions out of 23 induced an inhibition effect on microbial respiration, although only the fraction F11 was significant (-36%, *t* = 2.81, *P* = 0.047). Furthermore, the effect of *S. divinum* fractions varied over time [*F*_(__23__, 240__)_ = 1.77, *P* = 0.019]. Indeed, the fractions that had minimal effect after 6 h had a positive effect on microbial respiration after 21 h: F2 + 15% (*t* = 2.53, *P* = 0.045), F16 + 37% (*t* = 7.48, *P* < 0.001), F17 + 52% (*t* = 4.00, *P* = 0.019), F18 + 17% (*t* = 3.14, *P* = 0.021), F19 + 20% (*t* = 4.11, *P* = 0.008), F20 + 24% (*t* = 5.138, *P* = 0.004). Overall, 11 fractions out of 23 showed an inhibitory effect after 21 h of incubation, in particular the fractions F11 (−25%, *t* = 2.71, *P* < 0.05) and F9 (−6%, *t* = 2.81, *P* < 0.05).

### Effect of a Prolonged Allochthonous Leachate Addition on Microbial Food-Web Structure and Function

By the end of the 3 weeks leachate experiment, we did not find differences in CO_2_ respiration between the microbial communities that had been exposed every day to allochthonous leachate (L_*SD*_ and L_*SF*_ treatment) and those that had been watered only with rain water (C treatment). This result was apparent in the *S. divinum* microbiome [*F*_(__1__, 8__)_ = 1.22, *P* = 0.3] and the *S. fallax* microbiome [*F*_(__1__, 8__)_ = 2.68, *P* = 0.14; Supplementary Figure S4]. Similarly, we did not find any significant effect of treatment on overall enzyme activity (FDA) for both *Sphagnum* species (Supplementary Figure S5).

**FIGURE 4:**
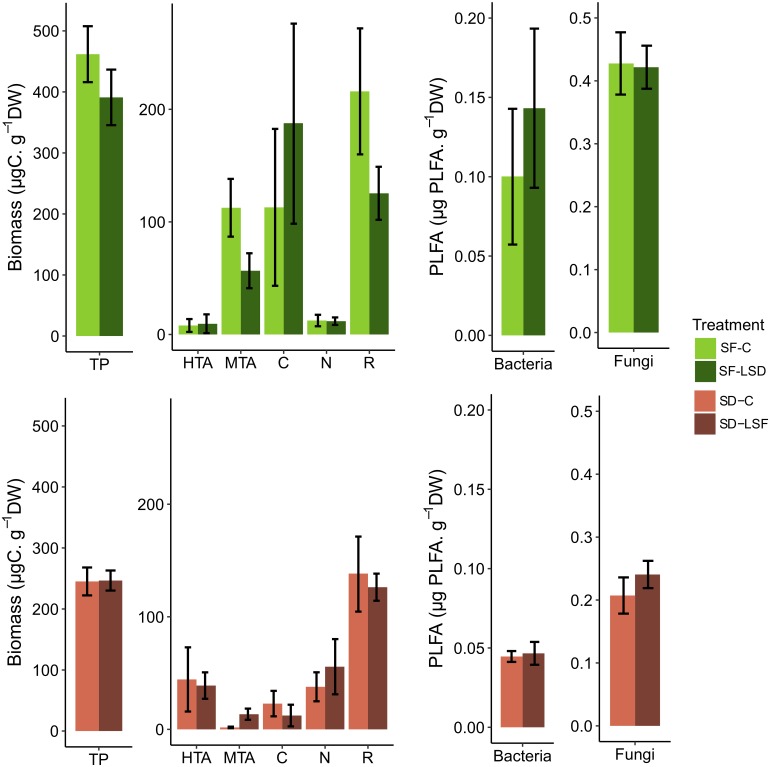
Structure of microbiome in *S. fallax* **(top)** and *S. divinum* **(bottom)** microcosms after 3 weeks of daily exposition to rain water (SF-C, SD-C) or allochthonous leachates (SF-L_*SD*_, SD-L_*SF*_). For each *Sphagnum* species, the two graphs on the left show the predator biomass with TP, total predators, i.e., the sum of HTA, heterotrophic testate amoebae; MTA, mixotrophic testate amoebae; C, ciliates; N, nematodes; R, rotifers. The two graphs on the right show the relative abundance of bacteria and fungi. No significant differences were detected. Error bars refer to standard error.

The analysis of the *Sphagnum* microbiome community composition showed a clear difference in terms of species diversity and species biomass between *S. fallax* and *S. divinum* microcosms. The first two axis of the PCA (Supplementary Figure S6) showed that *Hyalosphenia papilio* (mixotrophic testate amoebae), *Amphileptus* sp. (ciliates), *Lecane quadridentata* and *Polyarthra* sp. (rotifers) were dominating in *S. fallax* microbiome, while *Hyalosphenia elegans* (heterotrophic testate amoebae), *Colurella obtusa* (rotifers) and nematodes dominated the *S. divinum* microbiome. We found that 3 weeks of allochthonous leachate addition tended to alter the composition of microbial communities in *S. fallax* microcosms, but not in *S. divinum* microcosms (Figure 4). In the *S. fallax* microbiome, we observed a decrease in biomass of some predators, so that the biomass of testate amoebae decreased by 45% [*F*_(__1__, 8__)_ = 4.44, *P* = 0.07] mainly due to a decrease of mixotrophic testate amoebae [−50%, *F*_(__1__, 8__)_ = 3.63, *P* = 0.09]. Additionally, the biomass of rotifers decreased by 42% [*F*_(__1__, 8__)_ = 2.47, *P* = 0.16], although these trends were not significant.

**FIGURE 5:**
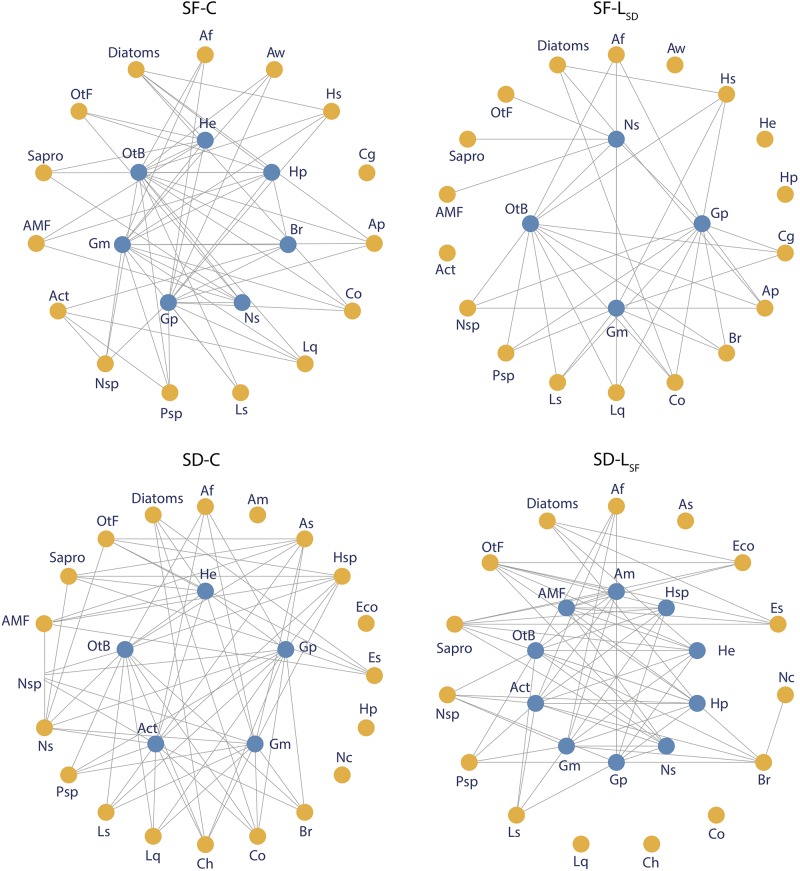
Food web structure in SF-C, SF-L_*SD*_, SD-C, and SD-L_*SF*_ microbiomes. Each point represent a microbial species or group and is colored according to its belonging to the core (blue) or to the periphery (yellow) of the network. Trophic links were weighted by the abundance of predators and the 20% weaker links were removed from the network. Abbreviations of names are specified in Supplementary Table S1.

**FIGURE 6:**
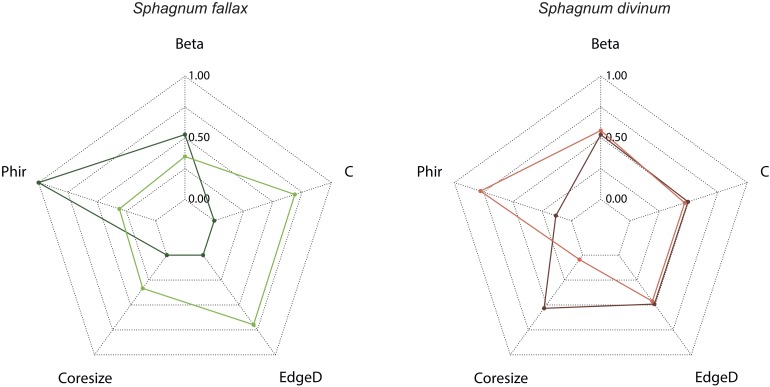
Structural indices of SF-C (left, light green), SF-L_*SD*_, (left, dark green), SD-C (right, light red), and SD-L_*SF*_ (right, dark red) food web networks. Structural indices comprised connectance (C), edge density within the network (EdgeD), core size, and edge density within the core (Phir). Beta diversity was also calculated with hypothetical networks. Each indice was normalized by its overall range.

The addition of allochthonous leachates caused changes in the food web structure, both in *S. fallax* and *S. divinum* microbiome (Figure 5). In the *S. fallax* microbiome, leachate addition led to a 30% decrease in connectivity and link density within the network (Figure 6). Similarly, the networks’ core size was reduced from seven species in the SF-C networks to four species in the SF-L_*SD*_ networks. Interestingly, two of the three species removed from the core were mixotrophs (*Hyalophenia papilio* and *Amphitrema wrightianum*). They were not only pulled away to the periphery of the cores, but they also lost the totality of their trophic links. We further found that SF-L_*SD*_ network was more different with the hypothetical network than SF-C network as network beta diversity was higher in the first case than in the second one (Figure 6). In *S. divinum* microbiome, we found an opposite effect of allochthonous leachate addition. In particular, we found that core size was doubled in SD-L_*SF*_ networks (10 species) compared to SD-C networks (five species). Two species, i.e., *Amphitrema muscorum* and *Hyalophenia papilio*, that did not have any link in the SD-C network were further integrated to the core. The increasing of core size led to a decreasing of edge density within the core (0.11 and 0.40 in SD-L_*SF*_ and SD-C networks respectively). Allochthonous leachate addition did not affect neither connectivity and edge density of the networks, nor network beta diversity.

The networks obtained after randomization and/or random species loss strongly differed from SF-L_*SD*_ and SD-L_*SF*_ networks, both in term of network beta diversity and structural indices (SES were strongly different from 0, *P* < 0.001, Supplementary Figure S7). This result was found both in *S. fallax* and *S. divinum* microbiomes, irrespective of the null models tested (Supplementary Figure S7). This indicates that leachate-induced shifts in food web structures are due neither to random changes in interactions nor to random species loss. Finally, the target removal of *H. papilio*, *H. elegans* or both species from SF-C network did not lead to an increased similarity with SF-L_*SD*_ network (Supplementary Figure S8). On the same way, the addition of hypothetical links of *A. muscorum, H. papilio* or both species in SD-C network did not lead to an increased similarity with SD-L_*SF*_ network (Supplementary Figure S8).

## Discussion

*Sphagnum* specialized metabolites have long been suspected to drive microbial processes in peatlands (Verhoeven and Liefveld, 1997; Fenner and Freeman, 2011). In this study, we not only show that *Sphagnum* specialized metabolites comprise a mosaic of microbial and *Sphagnum* compounds, but also that these complex assemblages trigger a set of important changes in the *Sphagnum*-microbiome structure and its functioning. We found that the effects of allochthonous *Sphagnum* leachate addition on competitive *Sphagnum*-microbiome were species-specific with antagonistic responses between *Sphagnum* species. While the addition of *S. divinum* leachates significantly reduced microbial CO_2_ respiration and altered the structure of *S. fallax*-associated food web, *S. fallax* leachate addition had only slight effects on *S. divinum* microbiome with no effects on microbial respiration. These results are even more striking by considering that both *Sphagnum* leachates mostly contain similar chemical compounds but in different proportions, and that the few *Sphagnum*-specific metabolites were poorly concentrated. Furthermore, we found that *Sphagnum* microbiomes strongly differed between species, suggesting that *Sphagnum* leachates target specific microbial species. Species-specificity has been shown as an important factor in plant-plant allelopathic interactions (Inderjit Wardle et al., 2011). Indeed, plant species are known to produce different cocktail of specialized metabolites according to their genus and environmental conditions, which can greatly influence their allelopathic interactions. Our results suggest that similar allelochemical mechanisms may exist between *Sphagnum* species and microorganisms. Although we cannot totally exclude that other factors than *Sphagnum* metabolites caused the observed differences in both *Sphagnum* microbiomes, the effect of *Sphagnum* metabolic fractions (Figure 3C) strongly suggests that *Sphagnum* metabolites can be a major driver of microbial CO_2_ respiration in peatlands.

### Microbial CO_2_ Respiration

Previous studies found that specialized compounds produced by *Sphagnum* mosses can inhibit bacterial growth (Mellegård et al., 2009) and microbial activity (Freeman et al., 2001; Fenner and Freeman, 2011). In this study, we show that such anti-microbial effects can be found in *Sphagnum* leachates but their magnitude is species-specific and their effects limited in time. Allochthonous leachate addition caused immediate decrease of *S. fallax* microbial respiration since the first exposure, while *S. fallax* leachate addition did not influence *S. divinum* microbial respiration. This result indicates that *S. divinum* leachates exhibit anti-microbial properties that promptly reduce catabolic activities of microbes associated to *S. fallax*, whereas the opposite is not the case. The analysis of *S. divinum* metabolic fractions on the respiration of *S. fallax* microbiome further demonstrates that not all *S. divinum* compounds inhibit microbial respiration. This result is supported by recent findings (Mellegård et al., 2009; Chiapusio et al., 2018) and suggests that anti-microbial effects of *Sphagnum* leachates depend both on metabolic cocktail and on the specific concentration of specialized metabolites (Chiapusio et al., 2018).

The general similarity in the composition of *S. divinum* and *S. fallax* leachates has, however, to be relativized by their low concentration. We cannot exclude that some compounds were under the limit of detection of the UHPLC-HRMS. Nevertheless, slight differences with potential important consequences were found in the composition of both leachates. For instance, sphagnum acid methyl ester – a derivate from sphagnum acid known for its anti-microbial effect (Verhoeven and Liefveld, 1997) was only found in L_*SD*_. Bacteria-related metabolites belonging to the hopane group of pentacyclic triterpenoids and associated to *Rhodopseudomonas* sp. (van Winden et al., 2012) were also only identified in L_*SD*_. Molecules of that group are known to have cytotoxic and antibacterial properties (Nagumo et al., 1991; Rohmer et al., 1991). This result is important and indicates that the metabolites secreted by *S. divinum*-associated bacteria could have negative effects on *S. fallax* microbiome. Although further research is needed to specifically identify the compounds with anti-microbial effects in the *Sphagnum*-sphere, our findings bring evidences that *Sphagnum* leachates result from the combined exudation and secretion of metabolites from *Sphagnum* and associated microorganisms. The composition of the resulting chemical assemblage is likely to determine leachate allelopathic potential, and might explain why *S. divinum* leachate has a higher allelopathic effect than *S. fallax* leachate.

Our results indicate that the inhibitory effect of *Sphagnum-*sphere specialized metabolites on microbial respiration in *S. fallax* microbiome is time limited and disappears after 2 days. Following this inhibition period, microbial respiration recovered at an important rate and the amount of CO_2_ respired overtook the one that was respired in control plots. This finding suggests that after the loss of inhibition, the nutrients and the DOC contained in *S. divinum* leachate might have stimulated microbial activity and respiration by supplying easily degradable C to microbes (Robroek et al., 2016). We found that prolonged addition of *S. divinum* leachates on *S. fallax* microbiome did not change microbial respiration despite structural, but functionally redundant changes among the microbial network. This result provides nuance to the traditional assumption that the accumulation of *Sphagnum* specialized metabolites such as polyphenols interferes with microbial catabolic activity in peatlands (Freeman et al., 2001). In our study, microbial enzyme activity and respiration seems to be resilient to prolonged accumulation of *Sphagnum* specialized metabolites due to a selection among microbial species. This suggests that *Sphagnum* leachates may play a role in natural microbial selection (Callaway et al., 2005; Inderjit Wardle et al., 2011) and that microbial adaptation within the community can lead to an alleviation of allelopathic effects (Li et al., 2015). Such alleviation effects might have important consequences for the peatland C balance and suggest that a critical re-examination of the mechanisms driven by *Sphagnum* specialized metabolites in peatland C dynamics are urgently needed.

### *Sphagnum* Microbiome and Microbial Networks

Our results show that *S. fallax* and *S. divinum* microbiomes differ and respond differently to allochthonous leachate addition to such an extent that a much stronger impact on *S. fallax* microbiome was observed compared to *S. divinum* microbiome. In both species, allochthonous leachate addition altered the food web structures, but in a different way. In *S. fallax* microbiome, L_*SD*_ altered the structure of the network through a decreasing of connectivity, edge density and core size. These alterations indicate a destabilization of the food web, which is reinforced by the fact that L_*SD*_ addition leads to a disparity with the hypothetical network. In fact, L_*SD*_ disrupts the intensity of trophic interactions between microbial species through a modulation of microbial abundance and, especially, a decrease of testate amoebae and rotifer abundance. More precisely, we found that mixotrophic testate amoebae, which combine both photosynthesis and predation, were dramatically isolated from the network in SF-L_*SD*_ treatments. This suggests that the duality in their nutrition does not provide them an advantage to resist to anti-microbial metabolites. It further indicates that *S. divinum* leachates may influence the survival and/or reproduction of mixotrophic testate amoebae, explaining why these species are poorly represented in *S. divinum* microbiome. García-Palacios et al. (2016) reported similar inhibitory effect of vascular plant polyphenols on specific microbial functional groups such as nematodes in soils. Our findings further point to a novel mechanism by which *Sphagnum* metabolites can have anti-predator effects, in addition to the already recognized anti-bacterial and anti-fungal effects (Verhoeven and Liefveld, 1997; Mellegård et al., 2009; Binet et al., 2017; Chiapusio et al., 2018). In the *S. divinum* microbiome, we found that *S. fallax* leachate addition also altered the food web structure, but with opposite effects compared to *S. fallax*. Particularly, we found that connectivity and core size increased in SD-L_*SF*_ treatment, which expresses a greater stability in the network (Csete and Doyle, 2004; Liu et al., 2011; Csermely et al., 2013). Therefore, it suggests that L_*SF*_ did not stress *S. divinum* microbiome but rather benefited and stabilized *S. divinum* associated food web. Those changes in the organization and the structure of *Sphagnum* food webs show that they are structurally unstable and that a perturbation such as allochthonous leachate addition can quickly change them toward a novel state with novel interactions and species. Simulated targeted removal or addition of the more impacted species in term of abundance or connectivity from control networks gave very dissimilar networks than those found with leachate addition. These findings suggest that, in addition to the direct loss or gain of trophic links, allochthonous leachates strongly modulate the trophic interactions among non-affected species through cascading effects. However, as mentioned above, the structural changes do not lead to important functional alterations of the microbial food web due to functional redundancies among microbes. These findings suggest that *Sphagnum* associated microbial food web functioning is somehow resistant to allochthonous *Sphagnum* metabolites thanks to structural plasticity. This further indicates that structural plasticity of microbial food webs tends to alleviate the effects of allochthonous leachate on its functioning on long-term. The underlying biochemical mechanisms still have to be elucidated further, but our results underline that notions of perturbation durations are primordial in the comprehension of their effect to peatland C dynamics.

### Implications for Competition

Plant competitiveness is intimately linked with plant fitness and allocation of resources to different metabolic processes (growth, reproduction, defense, etc.) (Herms and Mattson, 1992). In *Sphagnum*, plant fitness strongly depends on the associated microbiome since microbial activity drives nutrient cycling and production of plant-assimilable nutrients (Weston et al., 2015; Kostka et al., 2016). Hence, by their effects on microbial network structure and functions, allochthonous leachates probably alter *Sphagnum* fitness. For instance, the diminution of mixotrophic testate amoebae in *S. fallax* microbiome following *S. divinum* leachate addition could have decreased *Sphagnum* C uptake (Jassey et al., 2015). Further, the inhibition of microbial catabolic activity immediately after *S. divinum* leachate addition might have temporarily reduced nutrient availability within the *Sphagnum-*sphere. Over a longer time, the functional redundancy of microorganisms suggested that the microbial catabolic activities linked with C cycle were not affected by leachate addition. However, other microbial functions could have been impacted, especially those related to nitrogen or phosphate cycling (Carrell et al., 2019). This hypothesis is supported by Conkle and White (2012) who also found an immediate time-limited inhibition of microbial CO_2_ respiration by antibiotics in wetland soils with a durable alteration of microbial N_2_O respiration and phosphatase activity on longer term. As a consequence, an alteration of *Sphagnum* microbiome could impact *Sphagnum* competitiveness by affecting *Sphagnum* fitness. The increased leachate-resistance of *S. divinum* associated microbiome to allochthonous leachate suggests a better competitiveness than *S. fallax*. This result could have important consequences as it has been showed that hollow species (such as *S. fallax*) lose competitive strength under global warming (Breeuwer et al., 2008) so that the expected changes in *Sphagnum* distribution under global changes might be intensified by allelopathic mechanisms.

## Conclusion

Our findings have important implications for understanding how soil microbial communities respond to plant-associated chemical exudates. Although the exact nature of the metabolites interfering with microbial species still remains unknown, we refined the composition of *Sphagnum* leachates and extracts and showed that they can include microbial products. Further, our data show that addition of allochthonous leachates destabilize microbial network structure, primarily through anti-predators effects, with cascade consequences on microbial functioning and respiration. More research is needed to understand the exact genetic and physiologic mechanisms that define the observed response of microbial species to specialized metabolites. Our data reveal that microbiome alteration can be an important process in interspecific *Sphagnum* competition and indubitably in the C dynamics of peatlands. These findings suggest that facing the vegetation changes in peatlands due to climate warming and drought, specialized metabolites could significantly intervene in defense or offensive mechanisms through their effects on microbial communities.

## Author Contributions

VJ, BR, and SH designed and implemented the microcosm study with the help of CS. SH, BR, and VJ sampled the *Sphagnum* cores in the field and collected the leachates. SH and VJ collected the samples at the end of the experiment with the assistance of BR. SH performed MicroResp, characterized general characteristics of leachates and quantified enzyme activities with the assistance of VJ, BR, LB, and AB. VJ analyzed the microbial samples under the microscope. P-MA, SZ, and TS performed LC-MS analyses, peak data processing and molecular networks on *Sphagnum* leachates with assistance of GC and J-LW. SH and VJ performed statistical analyses and interpreted the data with assistance of BR, P-MA, LB, and AB. SH and VJ wrote the manuscript to which all authors contributed with discussions and text.

## Conflict of Interest Statement

The authors declare that the research was conducted in the absence of any commercial or financial relationships that could be construed as a potential conflict of interest.

## Footnotes

1 http://dnp.chemnetbase.com

2 http://gnps.ucsd.edu

3 www.cytoscape.org
