# Supplementary material for: Effects of Sphagnum Leachate on Competitive Sphagnum Microbiome Depend on Species and Time
Source: Front Microbiol. 2019 Sep 6;10:2042. doi: 10.3389/fmicb.2019.02042 (PMC6742715; doi:10.3389/fmicb.2019.02042)

Supplementary material

Effects of *Sphagnum* Leachate on Competitive *Sphagnum* Microbiome Depends on Species and Time

Samuel Hamard, Bjorn J.M. Robroek, Pierre-Marie Allard, Constant Signarbieux, Shuaihzen Zhou, Tongchai Saesong, Flore de Baaker, Alexandre Buttler, Geneviève Chiapusio, Jean-Luc Wolfender, Luca Bragazza, Vincent E.J. Jassey

This file contains one supplementary table and eight supplementary figures.

Note that supplementary figure S3 is provided in another file.

Table S1: Feeding habits of consumers and top predators found in *S. fallax* and *S. divinum* microbiomes.

			Feeding habit							
**Group**	**Species**	**Abbreviation**	**M**	**T**	**R**	**C**	**N**	**A**	**F**	**B**
**Testate amoebae**	*Amphitrema flavum*	Af	x							x
	Amphitrema wrightianum	Aw	x							x
	Assulina muscorum	Am							x	x
	Assulina seminulum	As							x	x
	Hyalosphenia sp.	Hsp							x	x
	Difflugia leidyi	Dl						x		
	Euglypha ciliata	Eci						x	x	
	Euglypha compressa	Eco						x	x	
	Euglypha strigosa	Es						x	x	
	Heleopera sphagni	Hs	x					x		x
	Hyalosphenia elegans	He						x	x	x
	Hyalosphenia papilio	Hp	x	x	x	x	x	x	x	x
	Nebela carinata	Nc		x	x		x			
	Nebela militaris	Nm						x	x	x
	Nebela tincta	Nt		x	x	x	x	x	x	x
**Ciliates**	*Cyclidium glaucoma*	Cg								x
	Amphileptus procerus	Ap		x				x		
	Paramecium bursaria	Pb	x					x		x
	Paramecium grosse sp	Pg						x	x	x
	Platyophrya sphagni	Ps	x							x
	Uronena sp	Us								x
**Rotifers**	*Bdeloïd rotifer*	Br								x
	Colurella obtusa	Co						x		x
	Coquille Habrotrocha	Ch						x		x
	Lecane ineris	Li								x
	Lecane quadridentata	Lq								x
	Lepedalla sp	Ls								x
	Polyarthra sp	Psp								x
	Trichocerca sp	Ts								x
**Nematodes**	*Nematode sp 1*	Ns							x	x
	Nematodes sp 2	Nsp								x

M = mixotroph; T = eating testate amoebae; R = eating rotifers; C = eating ciliates;

N = eating nematodes; A = algivorous; F = fungivorous; B = bacterivorous.

Figure S1: Number of links within SF-C, SF-L_{SD} (left), SD-C and SD-L_{SF} (right) networks in function of the threshold chosen to remove weaker links. We kept a threshold of 0.2, consisting in removing 20% of the weaker links.

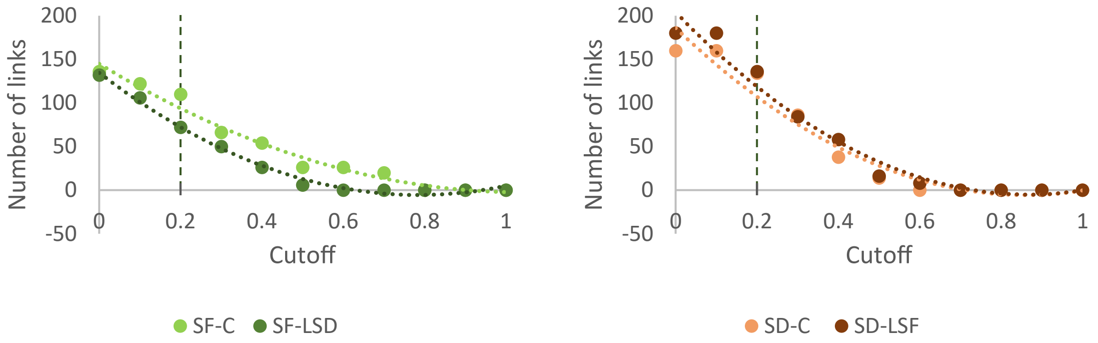

Figure S2: Clusters individualized from the massive similarity network (see Fig. S3) showing specialized metabolites predominant in L_{SD} (red) and with a minimal presence in L_{SF} (green) and bog water (blue).

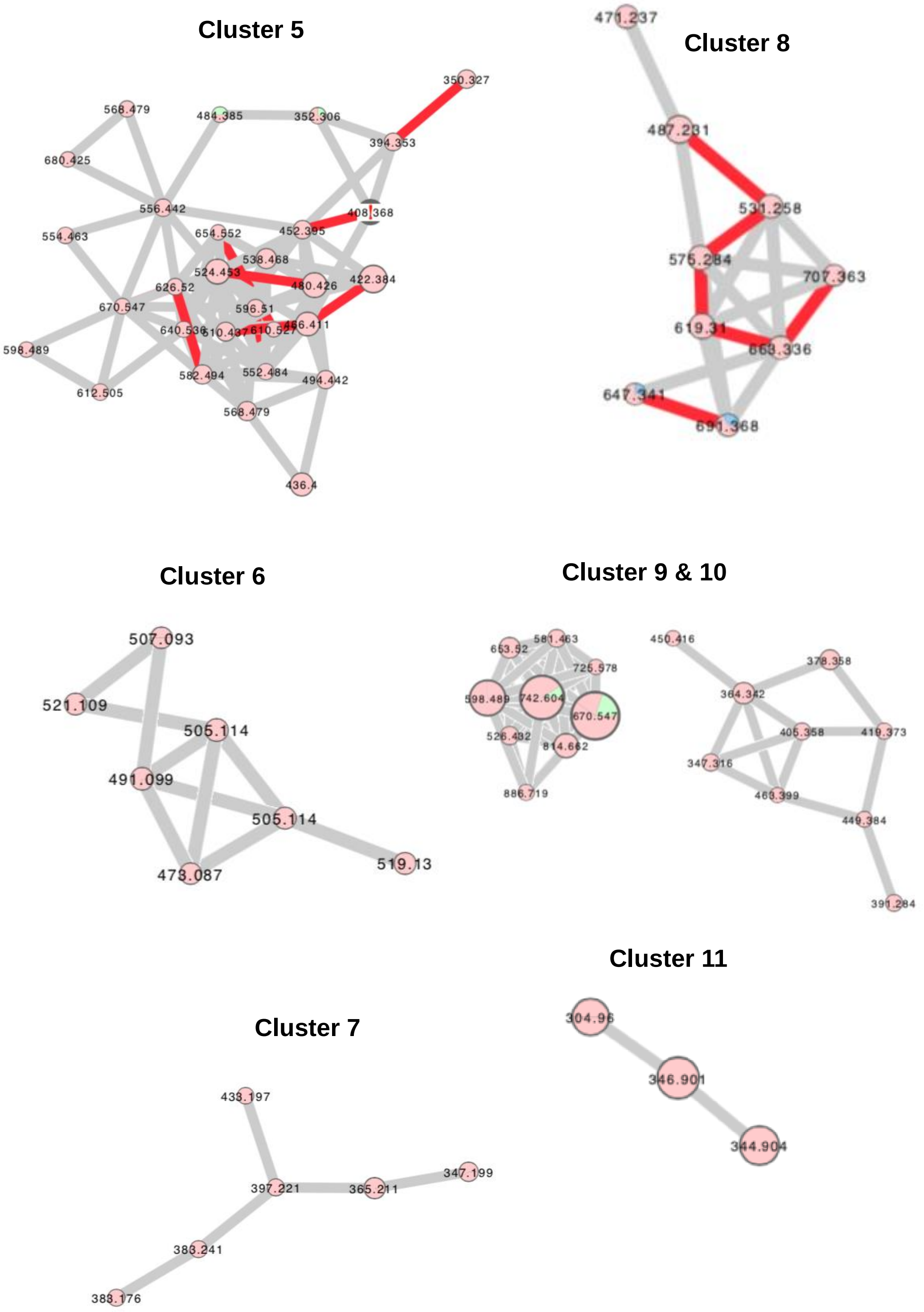

Figure S3: Massive similarity network of the metabolites found in *Sphagnum* leachates, methanolic and water extracts, and bog water, with positive (A) and negative (B) analysis.

The figure is provided in a separate file.

Figure S4: Respiration of *S. fallax* and *S. divinum* microbiome at the end of the experiment. The microbiomes from each experimental treatment (SF-C, SF-L_{SD} for *S. fallax* and SD-C, SD-L_{SF} for *S. divinum*) were incubated with water. Error bars refer to standard errors. No significant differences were found among treatments (ANOVA).

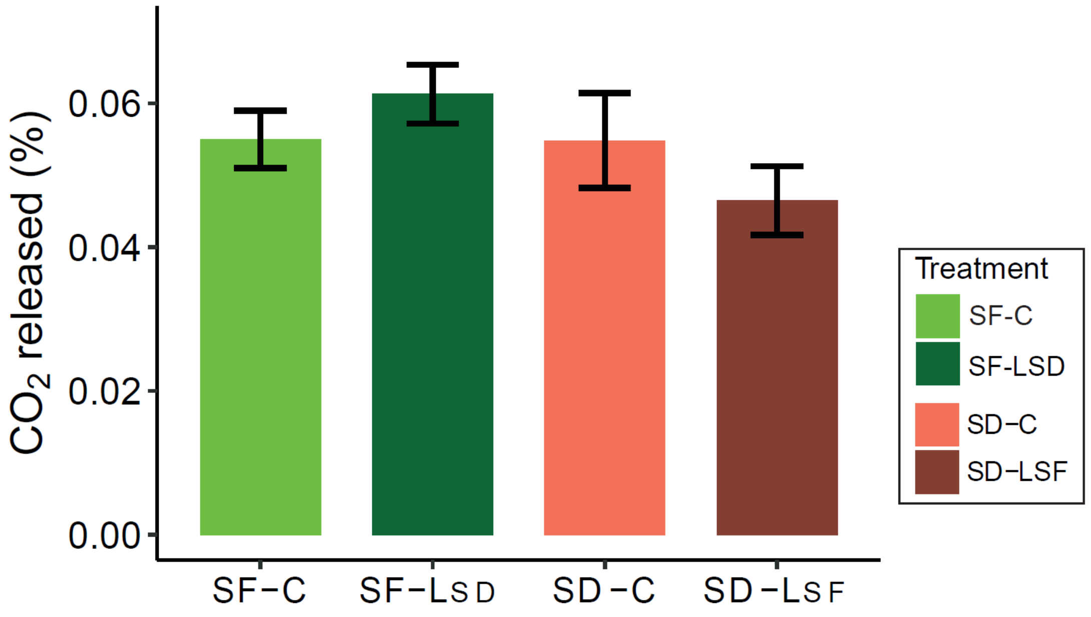

Figure S5: Overall enzyme activity (FDA) in *S. fallax* and *S. divinum* microcosms at the end of the experiment. Error bars refer to standard errors. No significant differences were found among treatments (ANOVA).

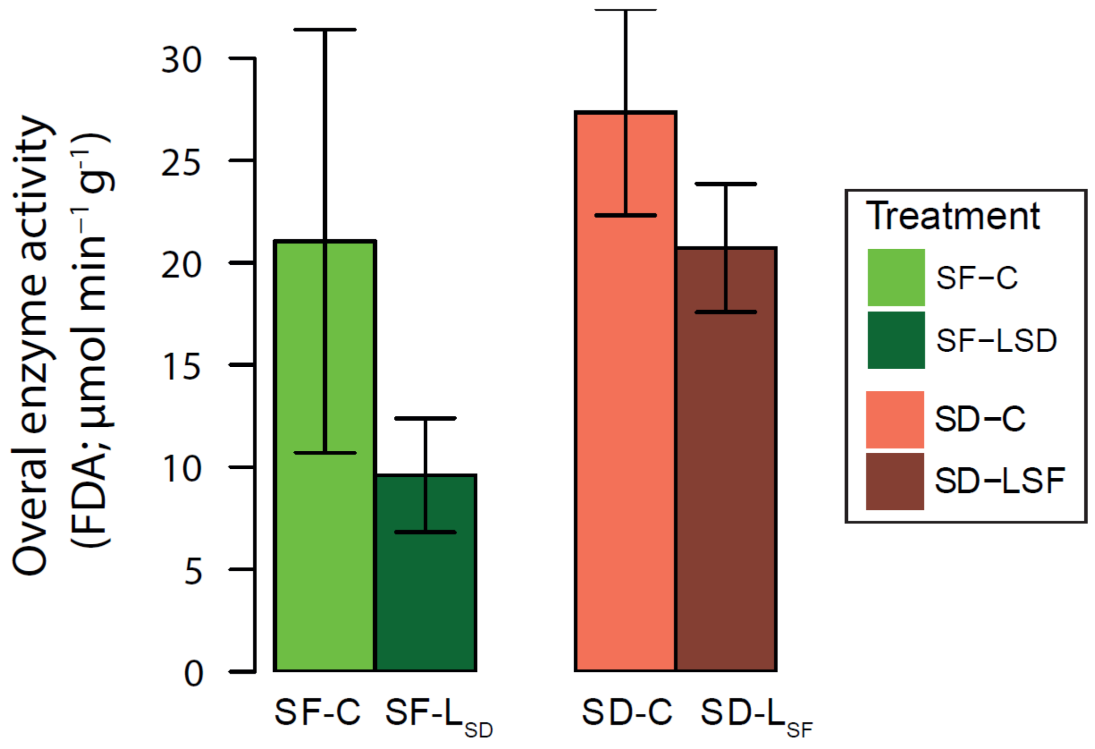

Figure S6: Principal component analysis of the different species of secondary consumers found in *S. fallax* (green circles) and *S. divinum* (red circles) microcosms after the three-week leachate experiment.

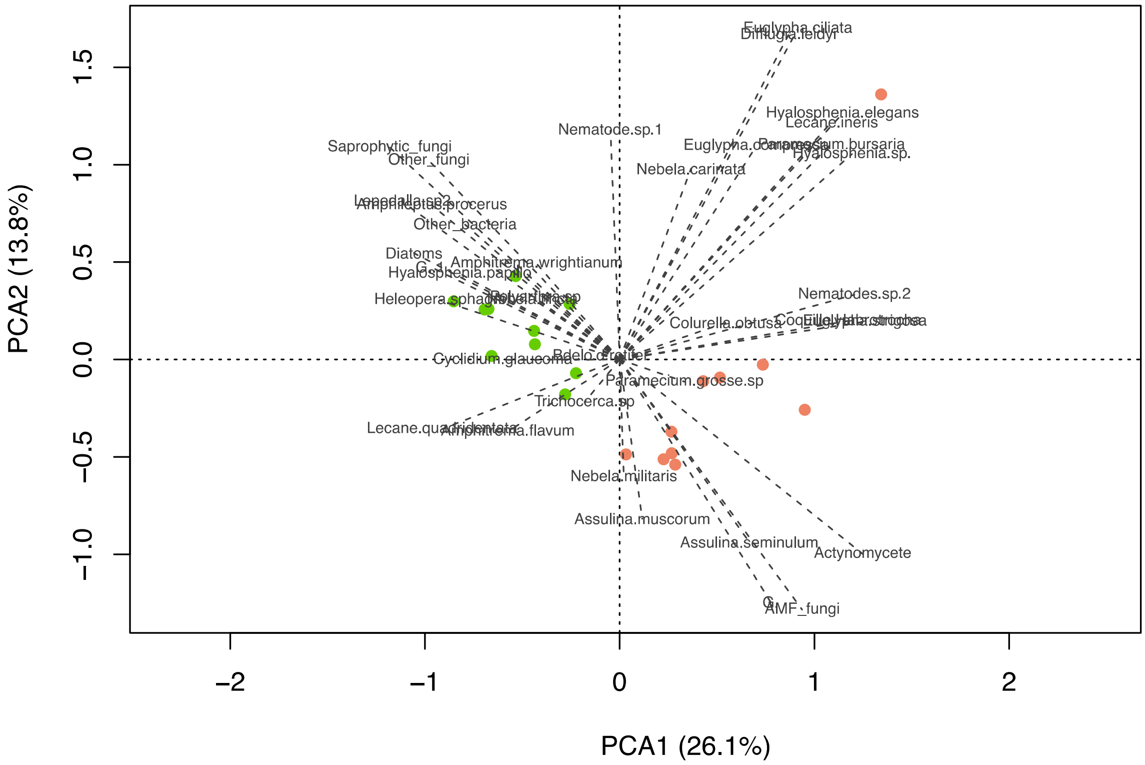

Figure S7: Differences between modelled trophic networks and leachate addition networks in *S. fallax* (green cirlces) and *S. divinum* (red squares) microbiomes, expressed as the deviation of the standardized effect sizes (SES) from 0. Modelled networks were simulated following three scenario (R_I, R_R, R_{IR}, see methods) and differences between networks were assessed on the five structural network indices: beta diversity with hypothetical networks, connectance (C), edge density within the network (EdgeD), core size, and edge density within the core (Phir).

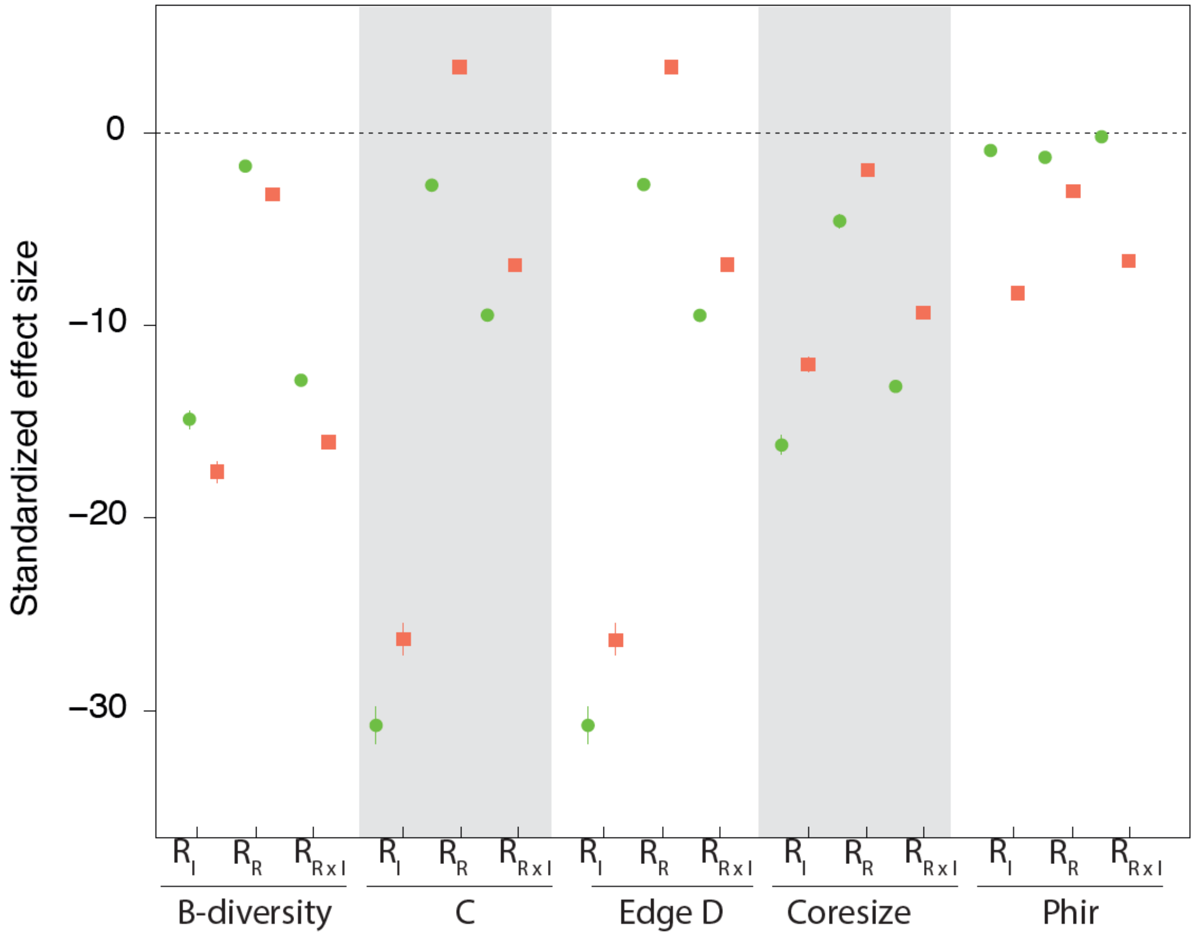

Figure S8: Structural indices in simulated networks constructed with a targeted removal or addition of key species from control networks. Simulated networks comprised the removal of *Hyalopshenia papilio* (-Hp), Hyalosphenia elegans (-He) and both species (-Hp -He) from SF-C network (left); and the addition of *Assulina muscorum* (+Am), *Hyalopshenia papilio* (+Hp) and both species (+Am +Hp) from SD-C network (right). Structural indices from Figure 6 were inserted on side graphs for comparison with the structural indices from networks of SF-C, SF-L_{SD}, (side graph on the left, light green and dark green respectively), SD-C and SD-L_{SF} treatments (side graph on the right, light red and dark red respectively).

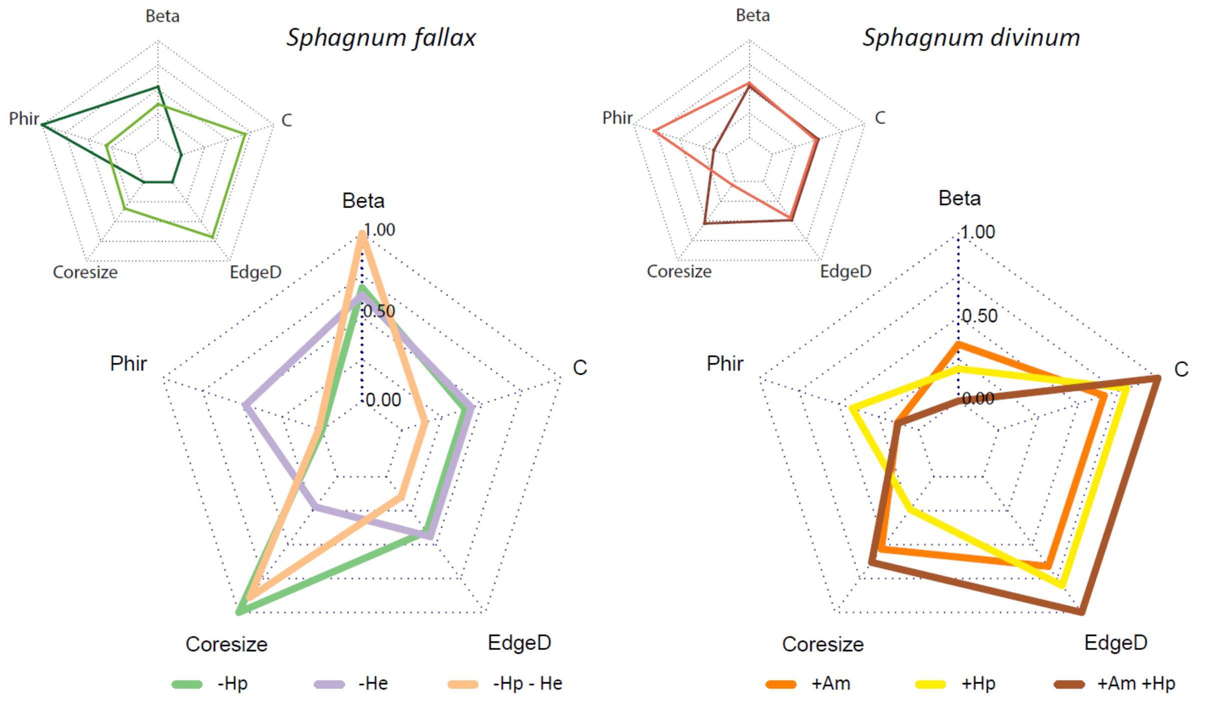

Supplement: Supplementary file 1 [file Data_Sheet_1.PDF]
