# Supplementary material for: Effects of Sphagnum Leachate on Competitive Sphagnum Microbiome Depend on Species and Time
Source: Front Microbiol. 2019 Sep 6;10:2042. doi: 10.3389/fmicb.2019.02042 (PMC6742715; doi:10.3389/fmicb.2019.02042)
Supplement: Supplementary file 2 [file Image_3.pdf]

**Figure S3:** Molecular network of the metabolites found in *Sphagnum* leachates, methanolic (MeOH) and water extracts ($H_2O$), and bog water, with positive (A) and negative (B) analysis.

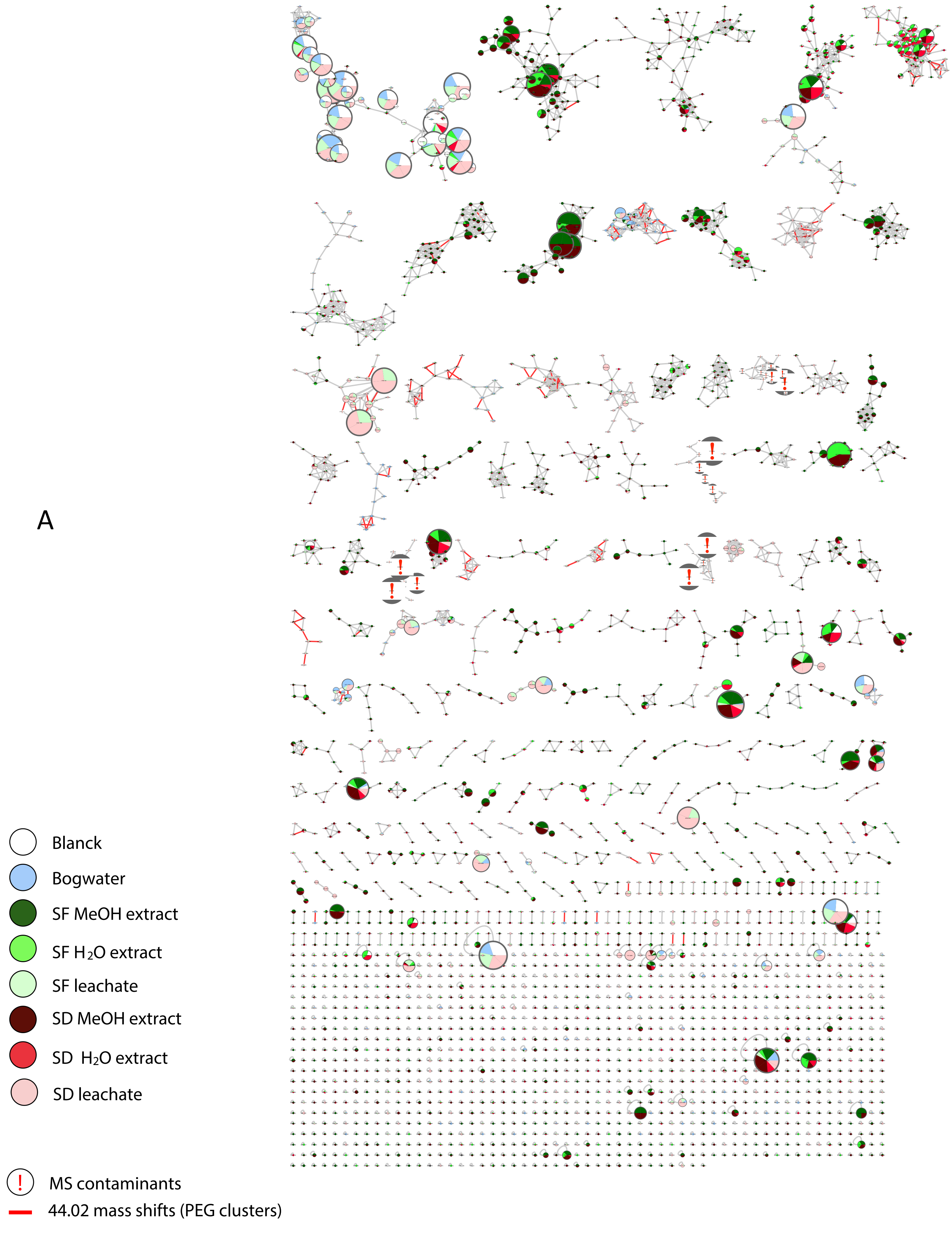

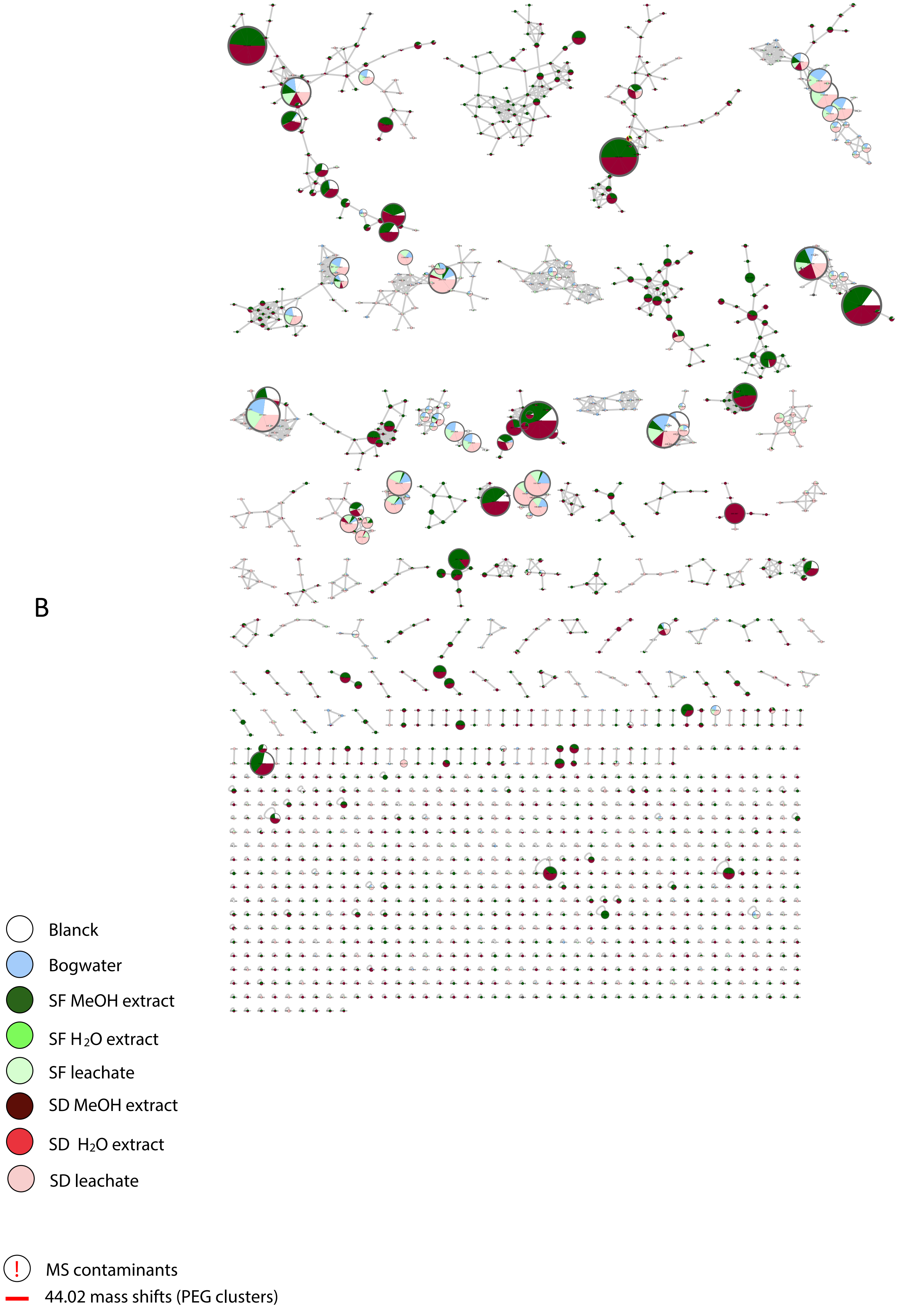
B
Blanck
Bogwater
SF MeOH extract
SF $H_2O$ extract
SF leachate
SD MeOH extract
SD $H_2O$ extract
SD leachate
!
MS contaminants
44.02 mass shifts (PEG clusters)
